# Supplementary material for: Overnutrition, Nasopharyngeal Pathogenic Bacteria and Proinflammatory Cytokines in Infants with Viral Lower Respiratory Tract Infections
Source: Int J Environ Res Public Health. 2022 Jul 19;19(14):8781. doi: 10.3390/ijerph19148781 (PMC9317356; doi:10.3390/ijerph19148781)
Supplement: Supplementary file 1 [file ijerph-19-08781-s001.zip › ijerph-1745218-supplementary.pdf]

Supplementary Materials

Supplementary Table S1. Association between severity of infection and presence of bacteria.

	Days of hospitalization		Days of oxygen therapy		Mechanical ventilation	
Explanatory variables						
Nutritional status	ON (n=47)	NW (n=69)	ON (n=47)	NW (n=69)	ON (n=37)	NW (n=69)
S. pneumoniae	1.15<br>(0.9-1.5)	1.27*<br>(1.0-1.6)	1.16<br>(0.8-1.5)	1.35*<br>(1.1-1.7)	-0.02<br>(-0.3-0.2)	0.2*<br>(0.1-0.3)
M. catarrhallis	1.36<br>(0.9-1.9)	1.04<br>(0.9-1.3)	1.35<br>(0.9-1.9)	1.04<br>(0.8-1.3)	-<br>-	-0.01<br>(-0.1-0.1)
H. influenzae	0.77<br>(0.5-1.1)	0.88<br>(0.7-1.0)	0.7<br>(0.5-1.1)	0.89<br>(0.7-1.1)	-0.21<br>(-0.6-0.2)	-0.02<br>(-0.2-0.1)
S. aureus	1.11<br>(0.8-1.5)	0.98<br>(0.8-1.2)	1.07<br>(0.8-1.5)	0.96<br>(0.8-1.2)	0.14<br>(-0.2-0.4)	<0.01<br>(-0.1-0.1)
Model	P	P	P	P	DP	DP

ON: overnutrition (obese and overweight). NW: normal weight. Cells show expected counts from Poisson models (P) or discrete change of dummy variable from Probit models (DP). -: variable was excluded from model because all individuals were positive for the bacteria. Cells also show, in parenthesis, the 95% confidence intervals. All regressions were controlled for gender, age, and a dummy variable including RSV infection alone, RSV-viral co-infections and no RSV infection. *p<0.05.

Supplementary Table S2. Association between severity of infection and bacterial load.

	Days of hospitalization		Days of oxygen therapy		Mechanical ventilation	
Explanatory variables						
Nutritional status	ON (n=47)	NW (n=69)	ON (n=47)	NW (n=69)	ON (n=37)	NW (n=69)
S. pneumoniae	1.03<br>(0.9-1.1)	1.04*<br>(1.0-1.1)	1.03<br>(0.9-1.1)	1.04<br>(1.0-1.1)	-0.01<br>(-0.02-0.01)	0.03*<br>(0.01-0.05)
M. catarrhallis	1.04*<br>(1.0-1.1)	1.02<br>(0.9-0.1)	1.05*<br>(1.0-1.1)	1.02<br>(0.9-1.1)	0.02*<br>(-0.01-0.01)	0.01<br>(-0.01-0.03)
H. influenzae	1.0<br>(0.9-1.1)	0.96*<br>(0.9-1.0)	0.98<br>(0.9-1.1)	0.96*<br>(0.9-1.0)	<-0.0<br>(-0.01-0.01)	-0.02<br>(-0.04-0.0)
S. aureus	1.06<br>(1.0-1.1)	1.0<br>(0.9-1.0)	1.05<br>(0.9-1.1)	0.99<br>(0.9-1.0)	0.01*<br>(-0.01-0.03)	<0.0<br>(-0.03-0.02)
Model	P	P	P	P	DP	DP

ON: overnutrition (obese and overweight). NW: normal weight. Cells show expected counts from Poisson models (P) or discrete change of dummy variable from Probit models (DP). Cells also show, in parenthesis, the 95% confidence intervals. All regressions were controlled for gender, age, and a dummy variable including RSV infection alone, RSV-viral co-infections and no RSV infection. *p<0.05.

Supplementary Table S3. Association between severity of infection and presence of >2 bacteria at the same time.

	Days of hospitalization		Days of oxygen therapy		Mechanical ventilation	
Explanatory variables						
Nutritional status	ON (n=47)	NW (n=69)	ON (n=47)	NW (n=69)	ON (n=37)	NW (n=69)
>2 bacteria at the same time	1.13 (0.9-1.5)	1.23 (1-1.5)	1.06 (0.8-1.4)	1.30* (1.03-1.05)	0.01 (-0.01-0.2)	0.19 (<0.0-0.4)
Model	P	P	P	P	DP	DP

ON: overnutrition (obese and overweight).; NW: normal weight. Cells show expected counts from Poisson models (P) or discrete change of dummy variable from Probit models (DP). Cells also show, in parenthesis, the 95% confidence intervals. All regressions were controlled for gender, age, and a dummy variable including RSV infection alone, RSV-viral co-infections and no RSV infection.*$p<0.05$.
